# Trees just go “nuts”: prioritizing carbon allocation to yield in almond trees

**DOI:** 10.1007/s00425-026-05003-0

**Published:** 2026-04-28

**Authors:** Shreya S. Veeravelli, Andrew J. McElrone, Ian R. Wright, Mina Momayyezi, Kyle R. Knipper, Nicolas E. Bambach, Sebastian Castro Bustamante, Andrew J. Gal, Sat Darshan S. Khalsa, Ranjith Karunakaran, Hung T. T. Nguyen, Morgan E. Furze

**Affiliations:** 1 Department of Botany and Plant Pathology, Purdue University, 915 Mitch Daniels Blvd, West Lafayette, IN 47907 USA; 2 Center for Plant Biology, Purdue University, 915 Mitch Daniels Blvd, West Lafayette, IN 47907 USA; 3 Department of Plant Sciences, University of California, Davis, Davis, CA 95616 USA; 4 Crops Pathology and Genetics Research Unit, USDA-ARS, Davis, CA 95616 USA; 5 Sustainable Agricultural Water Systems Unit, USDA-ARS, Davis, CA 95616 USA; 6 Department of Earth Science and Environmental Change, University of Illinois Urbana-Champaign, Urbana, IL 61801 USA; 7 Department of Forestry and Natural Resources, Purdue University, 915 Mitch Daniels Blvd, West Lafayette, IN 47907 USA

**Keywords:** Dormancy, Nonstructural carbohydrates, Perennial crop, *Prunus dulcis*

## Abstract

**Main conclusion:**

Almond trees prioritize C to yield rather than stem growth, and their overall lower NSC stores compared to forest trees may have consequences for resilience under environmental variability.

**Abstract:**

Perennial tree crops store nonstructural carbohydrates (NSCs) as energy reserves that can be used to persist during both predictable periods of reduced activity like dormancy and more unpredictable periods associated with stress. For deciduous tree crops, which lose their leaves at the start of dormancy, the NSC reserves accrued by that time are critically important for fueling respiration but may also influence processes in the following growing season. To quantify the seasonal NSC fluctuation surrounding dormancy and its influence on downstream processes like growth and yield, we conducted a comparative study of four almond varieties in a commercial orchard (California, USA). Sugar and starch concentrations were quantified in branch, stem, and coarse root when entering and exiting dormancy. We then assessed the correlation between these NSC data and metrics of stem growth and yield in the following growing season. We further explored long-term trade-offs between stem growth and yield using historical data from 2017 to 2022. Overall, total NSC concentrations significantly decreased during the dormant season in all organs. We observed a significant positive correlation between branch total NSC concentration when entering dormancy and yield the next year. However, we unexpectedly found that stem total NSC concentrations when entering dormancy were negatively correlated with stem growth the next year, suggesting that stem reserves were primarily used to support wintertime respiration or translocated to other organs. A long-term trade-off between stem growth and yield was evident; as yield increased, basal area increment tended to decrease. Additionally, we found whole-tree NSC storage to be lower in almond trees compared to temperate forest trees, reflecting prioritization to yield over NSC storage. Overall, these findings advance our understanding of crop tree carbon physiology and provide insight into the resilience of different almond cultivars under changing environmental conditions.

**Supplementary Information:**

The online version contains supplementary material available at 10.1007/s00425-026-05003-0.

## Introduction

Nonstructural carbohydrates (NSC) play a fundamental role in the overall physiological function of trees, serving as a source for growth, metabolism, and defense and allowing trees to survive both predictable (e.g., dormancy) and unpredictable (e.g., drought and pest outbreaks) events (Hartmann and Trumbore 2016). By investing a portion of photosynthates to storage as NSCs (i.e., primarily soluble sugars and insoluble starch), trees have the capacity to withstand periods of environmental stress, as well as support critical physiological and phenological processes, such as leaf out, flowering, and xylogenesis by accessing these reserves (Dietze et al. 2014; Hartmann et al. 2020). Thus, the persistence of long-lived tree species relies on sufficient accumulation of NSC reserves to support carbon (C) demands when photosynthesis is impaired or absent.

For deciduous trees, which lose their leaves in the autumn, the NSC reserves accrued by the start of the dormant season are critically important for downstream processes (Tixier et al. 2019). Not only do these NSC reserves support respiration throughout the dormant season when photosynthesis is absent, but they also influence processes in agricultural species that occur when trees exit dormancy—including growth, flowering, and yield (Maurel et al. 2004; Gough et al. 2009; Sperling et al. 2019; Tixier et al. 2020; Zwieniecki et al. 2022). Interestingly, while the seasonal dynamics of NSCs are thought to be driven by changes in phenology (Kozlowski 1992; Martínez-Vilalta et al. 2016), recent work suggests that NSC dynamics may also trigger phenological events (Blumstein et al. 2024; Luo et al. 2024). This suggests an additional role for NSCs in initiating the proper timing of phenological stages, but may also lead to physiological dysfunction if a disturbance alters typical NSC levels prior to the onset of key phenological stages (Roxas et al. 2021). Furthermore, the extent to which reserves are accumulated and remobilized throughout the year is species and organ dependent (Smith et al. 2017; Furze et al. 2018a, b). For example, at the whole-tree level, the size and seasonal fluctuation of reserves differs between deciduous species based on traits like wood anatomy (Barbaroux et al. 2003; Furze et al. 2018a, b), and at the organ-level, branches often have the largest seasonal fluctuations as their reserves are remobilized as the closest source to support leaf out and flowering (Bonhomme et al. 2005; Furze et al. 2018a, b; Fermaniuk et al. 2021). Overall, two critical time points emerge for assessing a deciduous tree’s NSC reserves in the context of preparedness for the dormant season and influence on post-dormancy processes—at the beginning and end of the dormant season. While previous studies in tree crop species have examined the relationship between seasonal NSC dynamics in twigs and yield (Zwieniecki et al. 2022) as well as seasonal NSC dynamics in above-ground organs and stem growth (Tixier et al. 2020), there exists an opportunity to explore these processes simultaneously and to further assess trade-offs over multiple years.

Like deciduous trees in natural systems, deciduous tree crops require stored NSCs to sustain plant functioning over the dormant season and successive phenological events post-dormancy. Unlike trees in natural systems where selection favors large NSC reserves as a safety buffer, there may be a lesser focus on building up NSC reserves in agricultural trees due to management practices selecting for more NSC to instead be directed toward maximizing yield (Zwieniecki et al. 2022). However, abiotic stresses, such as severe droughts and heatwaves, may increase irrigation demand, and this demand may not be fulfilled when coinciding with regulatory constraints on water use. Ultimately, abiotic stress and lack of water resources may negatively impact the C physiology and long-term health of perennial tree crops (Parker et al. 2020). For one, almonds, a deciduous nut tree crop, may change from their typical phenological activity with shifts in the timing of bloom and crop maturity. Projections to 2100 in the Central Valley (California, USA) suggest a shift to earlier flowering times, possibly due to the impact of dormant season temperatures on NSC dynamics (Sperling et al. 2015; Charrier et al. 2018; Orozco et al. 2024). Such shifts in season length and flowering time may make trees vulnerable to late frosts, cause pollinator mismatch, or further alter NSC storage and allocation patterns to disrupt post-dormancy processes. Thus, relating organ-level NSC reserves surrounding dormancy to subsequent growing season processes is useful for growers to understand the relative resilience of different varieties in the face of disturbances caused by environmental change.

To quantify the seasonal NSC fluctuation surrounding the dormant season and how these storage patterns influence post-dormancy processes in deciduous tree crops, we conducted a comparative study of four almond varieties [*Prunus dulcis* (Mill.) D.A. Webb cv. Butte, Carmel, Monterey, and Nonpareil] in an orchard setting (California, USA). Branch, stem, and coarse root tissues were analyzed for soluble sugar and starch concentrations when entering dormancy in November 2021 and when exiting dormancy in March 2022. Based on previous work in temperate forest trees (Furze et al. 2018a, b) and *P. dulcis* trees (Tixier et al. 2020), we expected that the two time points would reflect the maximum and minimum of NSC concentrations, respectively. We also expected NSCs to deplete between these two time points, and the extent of depletion to differ between organs, such that branch reserves would be the most dynamic. We then assessed the correlation between these NSC data with metrics of post-dormancy processes including stem growth and yield, as well as examined the potential trade-off between stem growth and yield. Understanding deciduous tree crop C physiology is important for identifying resilient crop tree varieties and improving orchard management strategies that aim to optimize tree health and yield.

## Materials and methods

### Site description

Almond trees [*P. dulcis* (Mill) D.A. Webb] were sampled from a 64.7-hectare (ha) commercial almond orchard located in CA, USA. The orchard is located in the Central Valley, a highly productive farming region which produces 80% of the world’s almond supply. The orchard contains four almond varieties that were planted in 2012 from approximately 2-year-old nursery stock on Bright Hybrid 5™ rootstock. The trees were spaced at 4.6 × 4.7 m and micro-sprinkler irrigated. The soil is clay loam (Kisekka et al. 2024).

The Nonpareil variety represents the majority of trees in the orchard, covering ~ 32.2 ha, as every other row was planted with this variety. The other three varieties—Butte, Carmel, and Monterey—are pollinizer varieties planted alternatively between each Nonpareil row. Since Nonpareil is not self-fertile, this variety requires compatible pollinizer varieties for effective pollination. The varieties at the orchard typically bloom in the following order: Nonpareil, Monterey, and Carmel, followed by Butte (pers comm.).

### Field collection

Study trees (*n* = 120 trees total; *n* = 30 trees per variety) were randomly selected across 23 rows in the orchard. We collected samples when entering dormancy in November 2021 and when exiting dormancy in March 2022 to capture the anticipated annual maximum and minimum NSC reserves, respectively. At both time points, branch, stem, and coarse root samples were collected for NSC analyses and stored on dry ice in the field and then at – 80 °C in the lab until processing. Multi-year branches (1–2 years old) were collected from the southwest side of each tree at mid-canopy using a hand pruner. Stemwood cores were collected from the south or southwest face of each tree using a standard 4.3-mm increment borer (Haglöf Company Group, Långsele, Sweden) attached to an electric drill. The bark was removed and the outer 3 cm (i.e., the metabolically active sapwood region) of the stemwood core was used for NSC analysis. Coarse root cores were collected in the same way as stemwood cores, but only the outer 2 cm of the root core was used for NSC analysis based on their smaller diameter.

In November 2022, two additional cores per tree (*n* = 40 cores total) were collected from a subset (*n* = 20 trees total, *n* = 5 trees per variety) of the 120 study trees for dendrochronological analysis. The cores were collected from the south and southwest face of each tree. Additionally, the diameter at breast height (DBH) was also recorded for all study trees at this time (Table S1).

### NSC analysis

Branch, stem, and root samples were dried at 100 °C for 1 h to denature starch degrading enzymes and then at 70 °C until completely dried. After drying, samples were ground on a Wiley mini mill (mesh 20; Thomas Scientific Wiley Mill, Swedesboro, NJ, USA). Approximately 30 mg of dried and ground tissue was weighed out for sugar and starch analyses following routine methods (Landhäusser et al. 2018).

Using hot 80% ethanol, bulk soluble sugars were extracted. The supernatant from the extraction then underwent a phenol–sulfuric acid reaction to colorimetrically determine the percentage of total sugar content. The bulk sugar extracts were read at a wavelength of 490 nm on a spectrophotometer (Thermo Fisher Scientific GENESYS 150 UV–Vis, Waltham, MA, USA). Sugar concentration (mg g^−1^, expressed as mg sugar per g dry weight of sample) was calculated from a 1:1:1 glucose–fructose–galactose (Sigma Chemicals, St Louis, MO, USA) standard curve.

After sugar analysis, we then used the dried tissue pellets for starch digestion with an *α*-amylase and amyloglucosidase enzyme solution. Glucose hydrolysate was determined using a peroxidase–glucose oxidase color reagent and read at 525 nm. Starch concentration (mg g^−1^, expressed as mg starch per g dry weight of sample) was calculated based on a glucose (Sigma Chemicals) standard curve. Total NSC concentration for each sample was determined by summing sugar and starch concentrations. For each individual batch of samples analyzed, at least one internal laboratory standard (red oak) was included to verify assay success and ensure that all values fell within ± 5% error of the long-term mean NSC values. The difference in sugar, starch, and total NSC concentration (*Δ*NSC) between time points was then calculated for each tree and organ as March 2022 values minus November 2021 values. NSC concentrations are provided in Table S1.

### Dendrochronological analysis

Stemwood cores (*n* = 40 cores total from 20 trees) collected for dendrochronology in November 2022 were air-dried and mounted on wooden blocks and prepared for tree-ring identification by sequentially sanding the cores. Cores were first sanded with a belt sander, using progressively finer grits of belts from 240 to 320. Cores were then hand sanded with grits of 600 and 800 and finished with grit 1000 for final polishing. Intermittent use of a USB-C digital microscope helped to determine ring boundaries/vessel clarity between stages of sanding. We determined the wood anatomy for our study trees to be semi-ring porous. Tree cores were imaged with digital resolution at 6400 dpi and optical resolution at 4800 dpi using an Epson Perfection V850 Pro scanner. Annual ring widths were measured to the nearest 0.01 mm using WinDENDRO software (Regent Instruments Inc., Canada).

We then used the annual ring widths to build a mean-value chronology for the site using the chron function in the dplR package in R (Bunn et al. 2025). The horizontal line method was selected for detrending along with a sample depth > 4 (which excluded 2015 only). The result was a ring width index (RWI) chronology for 2016–2022 for the site/orchard. A robust mean-value chronology was able to be established for the site, but not individual varieties due to limited cores (i.e., only *n* = 10 cores per variety).

Further, to assess a potential trade-off between annual stem growth increment and yield for each variety, we then used the annual ring widths to calculate the basal area increment (BAI) for 2017–2022 for each of the 20 trees cored for dendrochronological analysis. Yield data for 2016–2022 were obtained from orchard management. These data were provided for each variety at the orchard level (not individual trees) in net weight (kg ha^−1^), which refers to almond kernels only.

### Statistical analyses

To compare NSC concentrations between our four almond varieties, we used one-way analysis of variance (ANOVA) testing for each organ (branch, stem, or root) and NSC type (sugar, starch, or total NSC) to analyze concentrations among varieties for each time point independently (entering dormancy in November 2021 or exiting dormancy in March 2022). All models contained individual trees as a random effect. For significant models, differences between pairs of means were evaluated with Tukey’s honest significant difference (HSD) at *α* = 0.05.

Next, we sought to determine if there was a significant change in NSC concentrations between the start (November) and end (March) of dormancy in each organ for the four varieties (*Δ*NSC concentration = March 2022 concentration—November 2021 concentration). For each variety and organ, a one-sample *t* test was used to determine whether the mean *Δ*NSC concentration (sugar, starch, or total NSC) significantly differed from zero (*μ*_0_ = 0). Further, to determine if the extent of *Δ*NSC concentrations differed between varieties, we performed one-way ANOVA testing for each organ (branch, stem, or root) and NSC type (sugar, starch, and total NSC) to analyze *Δ*NSC concentrations among varieties. To determine if the extent of *Δ*total NSC concentrations differed between organs, we also performed one-way ANOVA testing for *Δ*total NSC concentrations among organs.

Further, we used a two-sided correlation test [with the cor.test() function in R] to examine the relationships between yield, growth, and NSC metrics. The relationships investigated are detailed in Table S5 due to different scopes of the data available for use (e.g., tree, variety, and orchard level). In brief, we assessed the relationship between organ-level NSC metrics and yield for 2022 at the variety-level, stem NSC metrics and BAI for 2022 at the tree level, yield, and BAI for 2017–2022 at the variety-level, and yield and RWI for 2016–2022 at the orchard level. Strength of association was evaluated using Pearson’s correlation at *ɑ* = 0.05. All data were checked for normality before analyses and all data analyses were performed in R version 4.4.1 (R Core Team 2021).

## Results

### NSC concentrations differed between varieties in above-ground organs, but not below-ground

In above-ground organs, total NSC concentrations significantly differed between varieties when entering dormancy (November 2021) and exiting dormancy (March 2022) (Figure S1C, F). In general, total NSC concentrations in the branches and stemwood tended to be lower in Nonpareil than the other varieties. For example, when entering the dormant season, Nonpareil branch concentrations were approximately 16% lower than Monterey and 10% lower than Butte. When exiting the dormant season, Nonpareil branch concentrations were ~ 10% lower than these same varieties. Similarly, Nonpareil stem concentrations were approximately 14% lower than Monterey and 10% lower than Butte when entering the dormant season, and approximately 10% lower than Butte when exiting the dormant season. In contrast to above-ground organs, total NSC concentrations in the roots did not significantly differ between varieties at either time point (Figure S1I).

### ΔNSC concentrations over the dormant season varied by organ

**Fig. 1:**
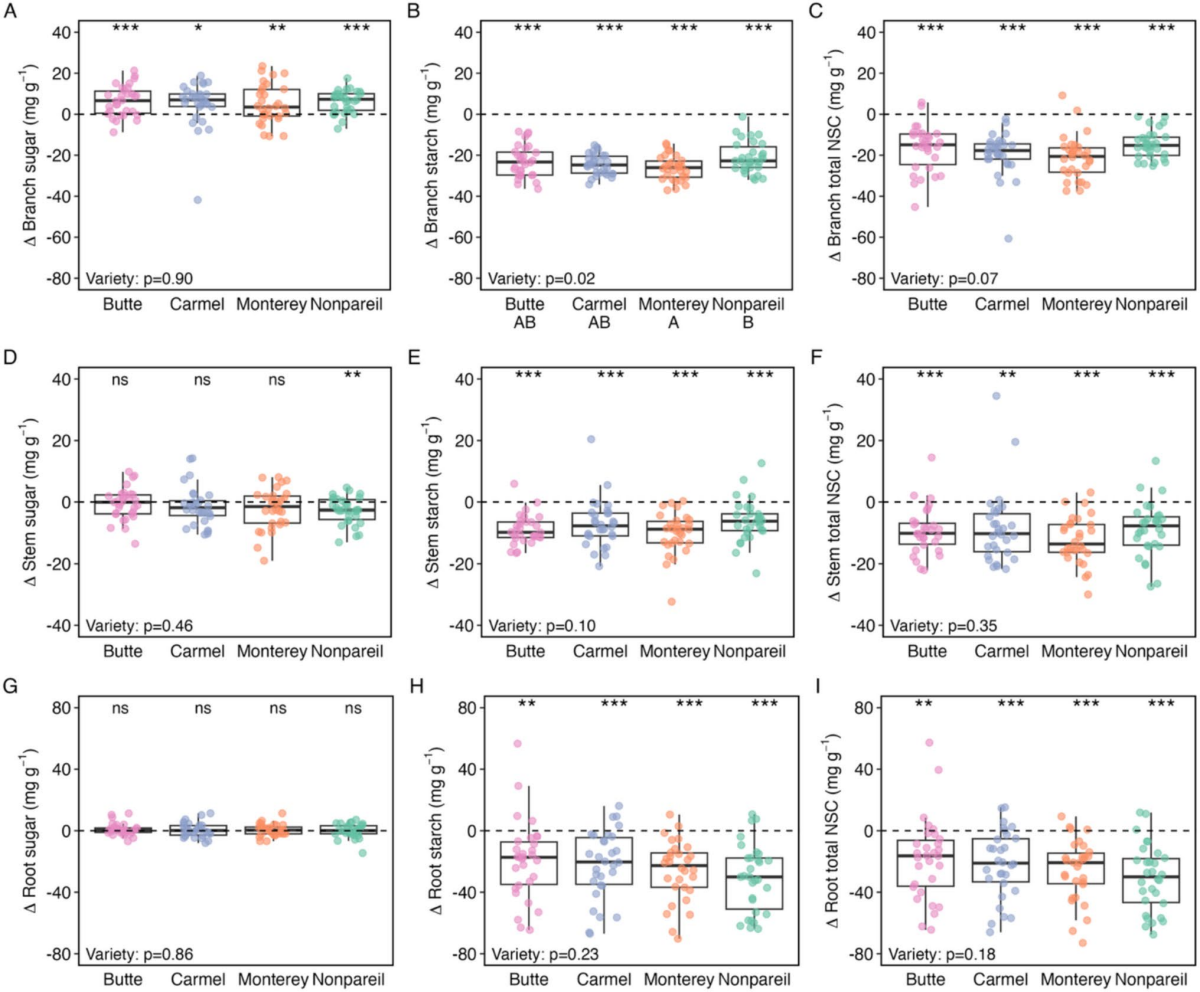
Change in sugar, starch, and total NSC concentrations (mg g^−1^) in the branches (**A**–**C**), stemwood (**D**–**F**), and roots (**G**–**I**) over dormancy in four almond varieties (Butte, Carmel, Monterey, and Nonpareil). *Δ*NSC concentrations were calculated as the NSC concentration when exiting dormancy (March 2022) minus the NSC concentration when entering dormancy (November 2021). Horizontal dashed lines are at 0, with points above the line indicating increased concentrations between the time points and points below the line indicating decreased concentrations between the time points. Each point represents an individual tree (*n* = 120 trees total), with each color representing a different variety (*n* = 30 trees per variety). The asterisks above each boxplot display the significance results of one-sample *t* tests; for each variety and organ, a one-sample *t* test was used to determine whether the mean *Δ*NSC concentration (sugar, starch, or total NSC) significantly differed from zero (*μ* = 0). *p* value < 0.001 = ***, *p* value < 0.01 = **, *p* value < 0.05 = *, and *p* value > 0.05 = ns. The *p* value displayed at the bottom left of each panel is that from a one-way ANOVA test for each organ and NSC type to analyze ΔNSC concentrations among varieties. Full statistical results are reported in Table S2, Table S3, and Table S4

In the branches, sugar concentrations significantly increased during the dormant season (Fig. 1A), while starch (Fig. 1B) and total NSC concentrations (Fig. 1C) significantly decreased (Table S2). In contrast, stemwood sugar concentrations did not significantly differ when entering and exiting dormancy, with the exception of a significant decrease between time points for Nonpareil (Fig. 1D). Stemwood starch and total NSC concentrations significantly decreased during the dormant season (Fig. 1E, F). Similar to the stemwood, root sugar concentrations did not significantly differ when entering and exiting dormancy (Fig. 1G), and root starch and total NSC concentrations decreased during the dormant season (Fig. 1H, I).

Overall, the change in concentrations did not significantly differ between varieties for any organ, except in the case of starch concentrations in branches. Branch starch concentrations tended to decrease over the dormant season to a lesser extent in Nonpareil compared to Monterey (Fig. 1B; Table S3). However, the change in total NSC concentrations did significantly differ between organs (Table S4). The mean change in total NSC concentrations across the dormant season was greatest for roots (– 24.1 mg g^−1^), followed by branches (– 18 mg g^−1^), and then stemwood (– 9.7 mg g^−1^).

### Relationship between NSC metrics and yield

**Fig. 2:**
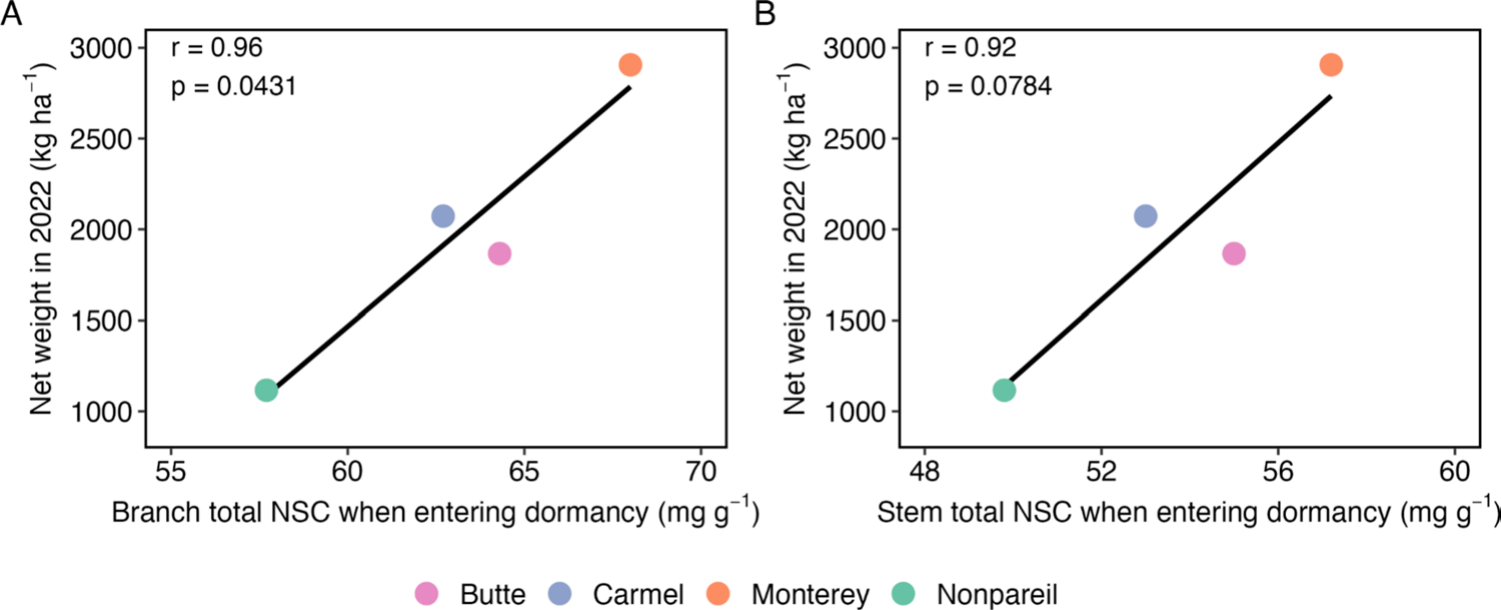
Relationship between yield and **A** branch total NSC when entering dormancy and **B** stem total NSC when entering dormancy. Each point and color represent a variety (Butte, Carmel, Monterey, and Nonpareil). Average yields were obtained for each variety (not individual trees) across the orchard, whereas branch and stem total NSC concentrations when entering dormancy were averaged across 120 study trees (*n* = 30 trees per variety). The Pearson’s correlation coefficient and *p* value of the two-sided correlation test [from the cor.test() function in R] are displayed in each panel. Strength of association was evaluated with Pearson’s correlation at *ɑ* = 0.05. Full statistical results are reported in Table S5

In the 2022 growing season following our NSC measurements, the varieties ranked as follows from largest to smallest yield: Monterey (2906.22 kg ha^−1^), Carmel (2072.80 kg ha^−1^), Butte (1866.15 kg ha^−1^), and Nonpareil (1116.32 kg ha^−1^). Unfortunately, individual tree yields were not recorded and only the mean yield for each variety was available for the orchard. We assessed the influence of organ-level NSC metrics on these yields. There was a significant positive correlation between total NSC concentration in the branches when entering dormancy and yield in the following growing season (Fig. 2A). While stem total NSC concentrations showed a similar trend, the correlation was not significant (Fig. 2B).

### Relationship between NSC metrics and stem growth

**Fig. 3:**
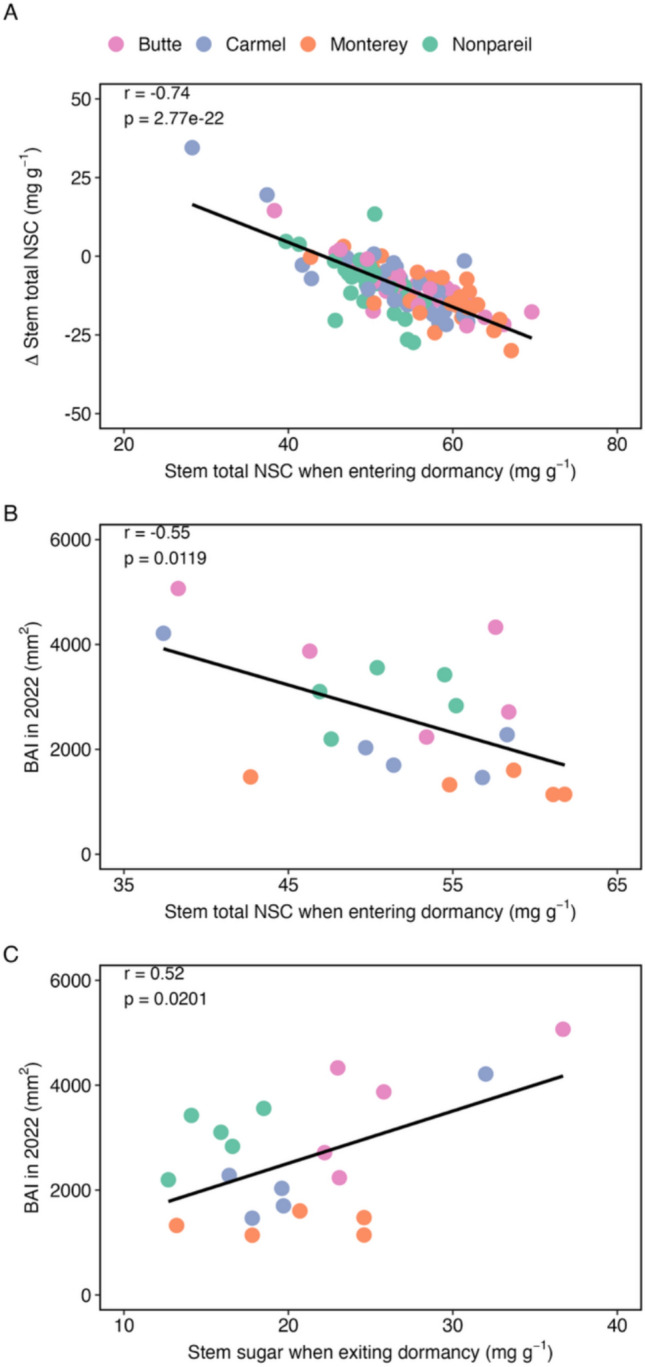
**A** Relationship between stem total NSC concentrations when entering dormancy and **B** the *Δ*total NSC concentrations in the stem over dormancy and **B** BAI the next year, as well as **C** the relationship between stem sugar concentrations when exiting dormancy and BAI that year. In **A**, each point represents an individual tree (*n* = 120 trees total), with each color representing a different variety (*n* = 30 trees per variety). *Δ*stem total NSC concentrations were calculated as the total NSC concentration when exiting dormancy (March 2022) minus the total NSC concentration when entering dormancy (November 2021). In **B** and **C**, a subset of the 120 study trees was cored for dendrochronological analyses. Thus, each point represents an individual tree (*n* = 20 trees total), with each color representing a different variety (*n* = 5 trees per variety). The correlation coefficient and *p* value of the correlation from the cor.test() function in R are displayed in each panel. Strength of association was evaluated with Pearson’s correlation at *ɑ* = 0.05. Full statistical results are reported in Table S5

As total NSC concentrations in the stemwood when entering dormancy increased, the change in total NSC concentrations over the dormant season increased (Fig. 3A). A reasonable assumption is that some of these local stem reserves may be going toward building the annual growth increment post-dormancy. However, our results suggest that higher stemwood total NSC concentrations at the start of dormancy corresponded to lower BAI in the following year (Fig. 3B); thus, we unexpectedly observed smaller BAI for trees with larger stem total NSC concentrations. Instead, we observed a positive correlation between stem sugar concentrations when exiting dormancy and BAI that year (Fig. 3C).

### Trade-off between yield and stem growth

We investigated historical stem growth to assess a potential trade-off with yield for a subset of the study trees (*n* = 20 trees used for dendrochronological analysis). The interseries correlation for the tree-ring series was 0.67 ± 0.31 (mean ± SD), which indicates that we could build a robust mean-value chronology at the orchard level (Figure S2). We also calculated BAI for each tree and obtained mean BAI values at the variety- and orchard-levels (Figure S3) to match the scope of yield data which was not available for individual trees, but rather for each variety at the orchard level. A representative image of a stemwood core used to calculate RWI and BAI is provided in Figure S2. From the mean-value chronology (Figure S2), RWI was greater than one from 2016 to 2019, indicating above-average growth. However, RWI was less than one from 2020 to 2022, indicating below-average growth. During this period, RWI dropped by over 60% from 0.95 to 0.50. This decrease was supported by BAI at the variety-level and orchard level (Figure S3).

**Fig. 4:**
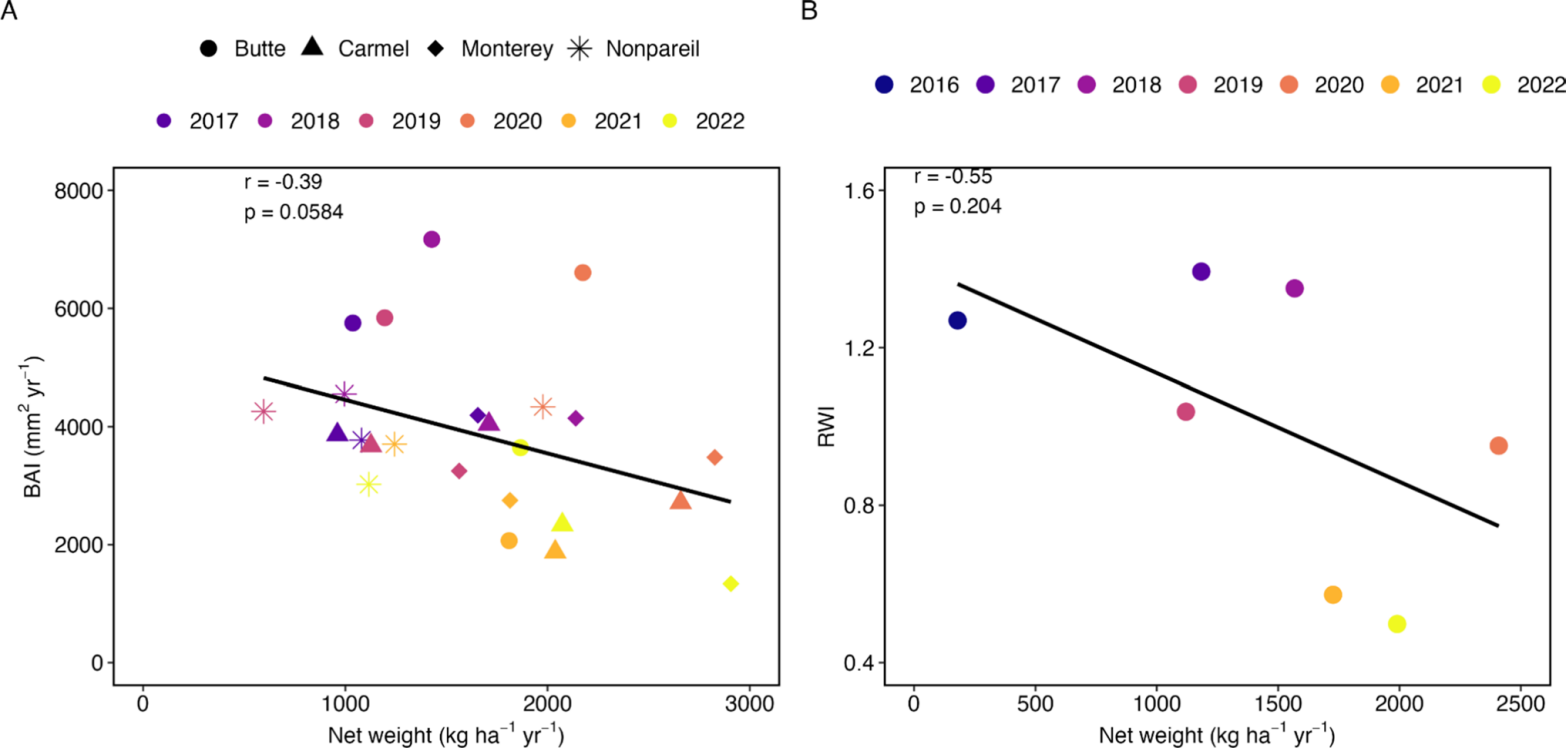
Relationship between yield and two stem growth metrics. **A** BAI from 2017 to 2022 and **B** RWI from 2016 to 2022. In **A**, each shape represents a variety and each color represents a year. Annual yields were obtained for each variety (not individual trees) across the orchard, whereas annual BAI was obtained by averaging across a subset of 20 study trees that were cored for dendrochronological analyses (*n* = 5 trees per variety). In **B**, each color represents a year. Since RWI values were obtained from a mean-value chronology constructed at the orchard level (Figure S2), the annual yield for the orchard was obtained by averaging the yields of the four varieties each year. The correlation coefficient and *p* value of the correlation from the cor.test() function in R are displayed in each panel. Strength of association was evaluated with Pearson’s correlation at *ɑ* = 0.05. Full statistical results are reported in Table S5

The mean-value chronology allowed for comparison between stem growth and yield at the orchard level for 2016–2022, whereas BAI allowed for comparison between stem growth and yield at the variety-level for 2017–2022. While both stem growth metrics tended to be negatively correlated with yield (Fig. 4), the correlation was only marginally significant for BAI (p = 0.0584). This suggested that as yield increased, BAI tended to decrease. Points toward the right side of the correlation plot corresponded to more recent years, indicating a temporal trend in the relationship between yield and BAI.

## Discussion

The start of dormancy marked by leaf fall and the end of dormancy marked by regrowth in the subsequent growing season represent two pivotal time points in the life cycle of deciduous tree crops. Between these time points, a series of physiological processes require C resources that are primarily supplied by previously stored NSCs in tree organs until photosynthesis resumes following dormancy and new photosynthate can then also contribute to these demands. We quantified NSC concentrations surrounding dormancy in the branches, stemwood, and coarse roots of four almond varieties to (1) compare NSC concentrations between varieties, (2) determine the extent to which NSC concentrations changed between the two time points in different organs, (3) assess the influence of NSCs on post-dormancy processes like stem growth and yield, and 4) examine the potential trade-off between stem growth and yield.

### NSC concentrations when entering and exiting dormancy

In all organs, total NSC concentrations when entering dormancy were largely reduced when exiting dormancy. The decrease across all organs is likely due to the fact that stored NSCs serve as substrates for respiration during the dormant season (Kozlowski 1992). Additionally, our post-dormancy sample collection occurred after flowering and leaf out as well as after stem growth had likely begun, and thus, these processes are expected to also be reflected in the observed drawdown of total NSC concentrations (Lebon et al. 2008). While depletion of NSCs over the dormant season has been observed in deciduous forest trees, with the largest fluctuations occurring in the branches due to their close proximity to newly emerging and developing leaves (Furze et al. 2018a, b; Fermaniuk et al. 2021), we found that total NSC concentrations were highly dynamic in both branches and roots. As soil temperatures begin to increase post-dormancy, root growth occurs prior to flowering in almond trees (Micke 1996), which aligns with our observed NSC depletion in the roots likely due to root development acting as a C sink during this time. Interestingly, the only organ which exhibited an increase in NSC concentrations over dormancy was branches, with higher branch sugar concentrations when exiting dormancy. Increased sugars in the branches may be a result of the conversion of starch to sugars to provide freezing tolerance to cells (Sauter et al. 1996; Kasuga et al. 2007; Charrier et al. 2015; Tarkowski and Van den Ende 2015) of exposed canopy branches in the dormant season. While freezing is not typically an issue at this California, USA study site, there was a freeze event in 2021 that may have impacted NSC dynamics. Further, since branch sugar concentrations were measured after leaf out occurred, it is possible that reallocation may have occurred to support bud development or current photosynthate may have begun to replenish branch sugars (Tixier et al. 2017; Roxas et al. 2021). However, previous work has shown that stomata on the leaves of red oak trees do not fully form until 1–2 weeks post-emergence (Kane et al. 2020), so it is possible that the stomata were not yet fully developed in our study trees at the time of branch sampling which was 1–2 weeks after budburst. Thus, increased branch sugars may not be from current photosynthate production.

Across all varieties, sugar and starch contributed roughly equally to total NSC concentrations in above-ground organs when entering dormancy, with sugars comprising 50.7 ± 0.7% of total NSC concentrations in the branches and 43.8 ± 0.9% of total NSC concentrations in the stemwood. However, when exiting dormancy, sugars increased to 84.3 ± 0.7% (mean ± SE) of total concentrations in the branches and slightly increased to 49.4 ± 0.9% in the stemwood. Seemingly, dormancy and/or the transition out of dormancy is characterized by the conversion of starch to sugars, further supporting the idea that sugars are integral for maintaining osmoregulation throughout the tree in the dormant season (Ögren 1997; Sperling et al. 2019). Concentrations of sugars toward the end of dormancy may also play a critical role in the timing of phenological events (Liu et al. 2021; Chen et al. 2022; Luo et al. 2024). In three Mediterranean nut tree species, including *P. dulcis*, twig NSC concentrations when entering dormancy impacted the timing of bud break and flowering (Roxas et al. 2021).

In contrast to above-ground organs, the fraction of total NSC concentrations comprised of starch was high in coarse roots when both entering (78.9 ± 0.5%) and exiting (67.3 ± 0.9%) dormancy. This is in line with the role of roots as a long-term storage organ with reserves that may serve as a critical buffer to stress (Loescher et al. 1990; Carbone et al. 2013; Shibata et al. 2016). Since root sugar concentrations did not differ when entering and exiting the dormant season, the depletion of starch concentrations in the roots may have helped to maintain sugar levels as well as fueled seasonal root growth and elongation (Noland et al. 1996; Contador et al. 2015). Thus, while previous work in five *P. dulcis* trees reported nearly identical levels and seasonal patterns of NSCs in the twigs, branches, and stemwood over the course of 16 months in 2015–2016 (Tixier et al. 2020), our findings from 120 trees sampled surrounding the dormant season in 2021–2022 highlight not only critical differences in NSC dynamics between above-ground and below-ground organs, but also among above-ground organs. Specifically, total NSC concentrations in the branches were nearly twice as dynamic surrounding dormancy compared to those in the stemwood.

### Relationship between organ-level NSCs and post-dormancy yield and stem growth

Previous work conducted in *P. dulcis* suggested that high post-dormancy NSC levels in February—immediately prior to and during flowering—were predictive of higher yields (Zwieniecki et al. 2022). Our results suggest a positive relationship between branch total NSC concentrations when entering dormancy (November) and next year’s yield. Our post-dormancy sampling month was March, not February. By March, a substantial amount of NSC reserves may have already been drawn down for processes local to branch reserves, such as leaf and flower development. Therefore, we may have found a positive relationship with our post-dormancy NSC concentrations if we had sampled earlier than March, just prior to these events. Further, we collected 1–2-year-old branches compared to their collection of current season twigs (Zwieniecki et al. 2022), which could impact NSC levels and their dynamics, since we captured older reserves integrated over multiple years. Also, our multi-year branch samples capture the location where nuts are primarily produced on older wood. Finally, perhaps, it is not surprising that we found that higher NSC levels when entering the dormant season suggest higher yields—if NSC levels are high at the onset of dormancy, and are maintained throughout dormancy or are restored/increased through translocation of sugars when exiting dormancy. These reserves can then be used to support flowering which ultimately links to yield.

While many previous studies have focused on NSC concentrations in the twigs of nut trees (Davidson et al. 2021; Zwieniecki et al. 2022), NSCs in the stemwood may also play an important role in supporting yield as the translocation of NSCs from distal organs such as the stem may be involved when exiting dormancy to supplement the use of branch NSC reserves stored prior to dormancy for leaf and flower development (Lacointe et al. 2004; Tixier et al. 2017). We found that as stemwood total NSC concentration when entering dormancy increased, next year’s yield tended to increase, but the correlation was not significant. We were only able to obtain yield data for each variety across the orchard which limited building this association with four values. Thus, future work should obtain yield data at the individual tree level to increase the sample size and further resolve the association between stem NSC metrics and yield.

Additionally, we showed that the higher the stem NSC concentrations when entering dormancy, the more was used during dormancy (Fig. 3A). Since our results suggested that stem NSCs were not significantly contributing to yield, we expected that a greater depletion of larger stem total NSC concentrations prior to dormancy would contribute to increased stem growth. Interestingly, the opposite was observed, and we found that higher stem total NSC concentrations when entering dormancy were associated with lower BAI the next year. This suggests that almond cultivars allocate NSCs to sinks other than stem growth. The majority of the stemwood NSCs depleted over the dormant season are likely used as substrates to fuel respiration (Sperling et al. 2015) or may be transported out of the stem to support bud growth and yield (as described earlier). Distal transport via the phloem is optimal at specific concentrations (Jensen et al. 2013); therefore, NSCs may have first accumulated to a specific threshold to allow transport to occur to distal sinks in the absence of transpiration in the dormant season and during the early growing season (Zwieniecki et al. 2015; Sperling et al. 2017).

However, stemwood sugar concentrations when exiting dormancy were positively associated with BAI, suggesting an important role for local sugars that are immediately available to support new ring growth rather than put into storage (Deslauriers et al. 2009; Furze et al. 2018a, b). In this study, we identified the wood anatomy of the four *P. dulcis* cultivars as semi-ring porous. Ring porous species initiate radial stem growth prior to leaf out and rely initially on previously stored reserves (Bréda and Granier 1996), further supporting the connection between stemwood sugars when exiting dormancy and stem growth that we observed. In *P. dulcis* grown in a managed orchard, stem growth began around bud break and ended during stages of fruit drop (Tixier et al. 2020). The almond cultivars in our study formed multiple bands of vessels throughout the growing season, likely coinciding with irrigation events. There may be an added NSC cost to support the formation of vessels over tracheids (Hacke and Sperry 2001; Olson et al. 2020).

### Trade-off between historical yield and stem growth

We assessed the potential trade-off between yield and stem growth by using two metrics of stem growth: RWI from the mean-value chronology constructed at the orchard level and BAI for individual trees. However, we only had average yield data across the orchard for each variety, not individual trees. Notably, the mean-value chronology showed a decline in RWI for 2020–2022 that suggested below-average growth for trees in the orchard, and this decline was supported by BAI measurements. We suspected that management practices like pruning may have shifted C allocation away from stem growth, since less structural support would be required to support a smaller canopy. However, pruning has been minimally used at our study site.

Nevertheless, as yield increased, BAI tended to decrease over the years from 2017 to 2022, with more recent years (2020–2022) falling out on the right side of the line in the region with the highest yields and lowest BAI. Over the same time period, leaf area index did not significantly differ between years (Fig. S4) (Houborg and McCabe 2018), suggesting that the decrease in stem growth was not due to increased allocation of C to canopy biomass over time. Further, the state of California experienced drought from 2020 to 2022 (Medellín-Azuara et al. 2022), so it is possible that trees could have been water-stressed during this time period, leading to reduced stem growth metrics. However, analysis of the Evaporative Stress Index (ESI), an agricultural drought indicator based on anomalies in the ratio of actual to reference evapotranspiration (Anderson et al. 2016), revealed no more negative trends during the 2021–2022 drought period than in 2018–2020 or 2023–2024, suggesting that irrigation was sufficient to prevent tree stress (Fig. S5). Historically, almond cultivars in California were bred and managed for yield (López-López et al. 2018; Lapsley et al. 2024), and in the four varieties in our study, C was prioritized for yield. Water stress can increase flowering as a survival mechanism (Takeno and Raines 2016), but it can also reduce nut weight and yield in subsequent growing seasons (Egea et al. 2010); thus, while drought stress during our study period was not an anomaly compared to other recent years, the increasing frequency and intensity of droughts may disrupt C allocation and threaten almond yield in the future.

### NSC differences between almond cultivars and the connection to yield

Above, we provided evidence for a positive association between total NSC concentrations in the branches when entering dormancy and next year’s yield. Monterey had the largest branch concentrations and Nonpareil had the smallest branch concentrations when entering dormancy, and this corresponded with the highest yield and lowest yield in each variety, respectively. However, some trees at the study site may be exhibiting alternate bearing patterns, as Nonpareil’s yield increased by nearly 2.5 times in the year after we conducted this study (pers comm.). Trees that exhibit alternate bearing patterns may accrue lower NSCs in the year prior to lower yields; however, such patterns are inconsistent between sites and cultivars (Tombesi et al. 2011). Overall, self-fertile Butte trees had comparable NSC stores and yields to the other three self-incompatible varieties. The use of self-fertile varieties in orchards eliminates the dependence on pollinators and bloom times with other compatible cultivars (Gradziel 2022), and thus, our physiological data may benefit growers looking to transition to self-compatible cultivars. As an aside, there was no difference in below-ground NSC concentrations between varieties, likely because all four varieties were grown on the same clonally propagated rootstock.

### Implications for the resilience of almond trees

Deciduousness, whether in an agricultural or forest tree species, forces trees into dormancy which can be a vulnerable period whereby energy reserves for continued function are restricted to previously stored NSCs. These reserves not only fuel respiration during the dormant season, but also our results demonstrate their influence on post-dormancy physiological and phenological processes. The extent to which NSCs are stored and used may have implications for the ability of trees to withstand unpredictable environmental stress. In agricultural tree crops like almonds, management practices may select for maximizing yield rather than maximizing NSC storage, whereas in natural systems, trees may build up larger reserves as a safety buffer. In temperate and tropical forest trees, NSCs have been estimated to comprise 4% and 8% of the biomass, respectively (Würth et al. 2005; Furze et al. 2018a, b). Using allometric equations, we estimated that NSCs make up between 0.78 ± 0.38% and 2.2 ± 0.9% (mean ± SD) of the biomass of the almond trees in our study, depending on which allometric equations are used (Jenkins et al. 2003; Chojnacky et al. 2014) (see Methods S1). Thus, almond trees are operating on lower NSC reserves compared to forest trees, which may have implications for their overall resilience in variable environments. Although management practices like fertilization, irrigation, and pruning can lessen the dependence on NSC reserves, under scenarios where these resources (i.e., water) are limited and costly, trees may become vulnerable.

## Conclusion

Herein, we have constructed a detailed assessment of NSC stores in almond trees by sampling at two time points surrounding dormancy, in major organs, and across four varieties. We then evaluated the association between these NSC metrics with yield and stem growth, as well as the potential trade-off between yield and stem growth. Rarely, there is a singular C source with a singular corresponding C sink (Hartmann and Trumbore 2016; Fatichi et al. 2019), highlighting the importance of interpreting NSC concentrations in the context of a range of processes that occur in different tree organs. Our knowledge of almond tree C dynamics has been limited due to past studies which explored associations between NSCs, growth, and/or yield in single species, single organs, and with small tree sample sizes. Our results, therefore, improve our understanding of C dynamics at both the organ- and whole-tree levels in almonds, and, importantly, suggest that their prioritization of C to yield rather than stem growth as well as their lower NSC stores compared to forest trees may have consequences for their ability to respond to changing environmental conditions. Future work should aim to quantify NSC consumption dynamics in relation to fruit set for additional perennial tree crops across diverse agroecosystems.

## Supplementary Information

Below is the link to the electronic supplementary material.

Supplementary file1 (PDF 3792 KB)

## Data Availability

Data in this manuscript are provided in the Supplementary Data file. Additional experimental data and materials are available upon reasonable request.
