# Supplementary material for: Trees just go “nuts”: prioritizing carbon allocation to yield in almond trees
Source: Planta. 2026 Apr 28;263(6):142. doi: 10.1007/s00425-026-05003-0 (PMC13124957; doi:10.1007/s00425-026-05003-0)
Supplement: Supplementary file 1 — Supplementary file1 (PDF 3792 KB) [file 425_2026_5003_MOESM1_ESM.pdf]

Supplementary Data

Trees just go “nuts”: prioritizing carbon allocation to yield in almond trees. *Planta*.

Shreya S. Veeravelli, Andrew J. McElrone, Ian R. Wright, Mina Momayyezi, Kyle R. Knipper, Nicolas E. Bambach, Sebastian Castro Bustamante, Andrew J. Gal, Sat Darshan S. Khalsa, Ranjith Karunakaran, Hung T.T. Nguyen, Morgan E. Furze*
*Corresponding author: mfurze@purdue.edu
Department of Botany and Plant Pathology, Purdue University, West Lafayette, IN, USA 47907
Center for Plant Biology, Purdue University, West Lafayette, IN, USA 47907
Department of Forestry and Natural Resources, Purdue University, West Lafayette, IN, USA 47907

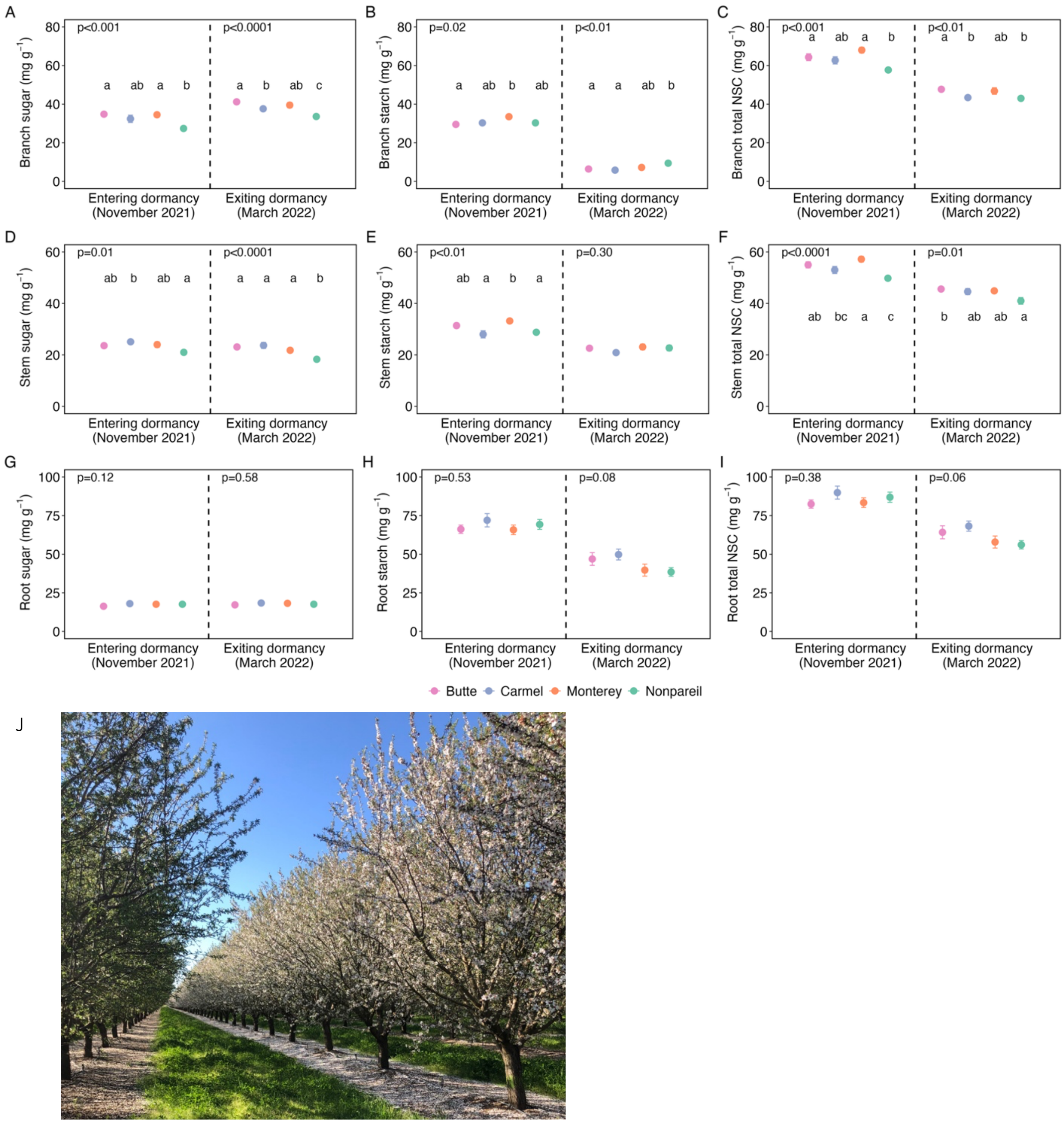

Figure S1. Sugar, starch, and total NSC concentrations (mg g^{-1}) in the branches (A-C), stemwood (D-F), and roots (G-I) of four almond varieties when entering dormancy (November 2021) and exiting dormancy (March 2022). Image of almond orchard in J. Error bars denote ± SE of the mean, and the error bars are smaller than the data points in many cases. Each mean was calculated from 30 trees. Color represents each variety. For each organ and NSC type, one-way ANOVA testing was performed to analyze concentrations among varieties for each month independently and the p-values for each are displayed. When significant, differences between pairs of means were evaluated with Tukey's honest significant difference (HSD at, α = 0.05, and lowercase letters indicate significance of differences among varieties within each month. Note the difference in y-axis scale between rows.

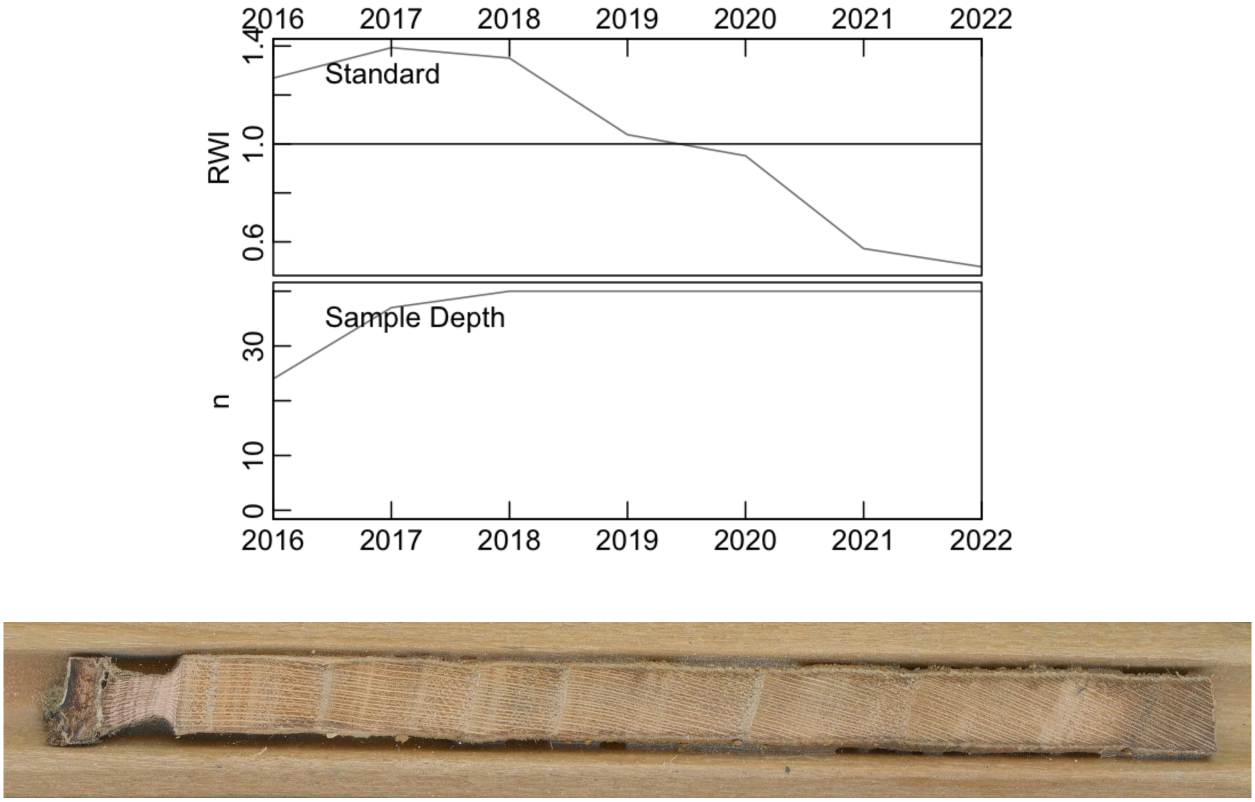

Figure S2. Mean-value chronology (top) constructed for the orchard. Cores were collected for dendrochronological analysis from a subset of study trees (n=20 trees total, n=5 trees per variety) in November 2022. Annual ring widths were obtained to build the mean-value chronology for the site using the chron function in the dplR package in R. The mean interseries correlation of the cores was 0.67. The horizontal line method was selected for detrending along with a sample depth >4(which excluded 2015 only). In the top panel, RWI is unitless and indicates average growth with values above 1 indicating above average growth and values below 1 indicating below average growth. In the bottom panel, sample depth gives the number of series available in that year. Representative image of a stemwood core (bottom) from a study tree that was used to calculate RWI and BAI. Additionally, the image shows the semi-ring porous wood anatomy of the almond varieties in this study.

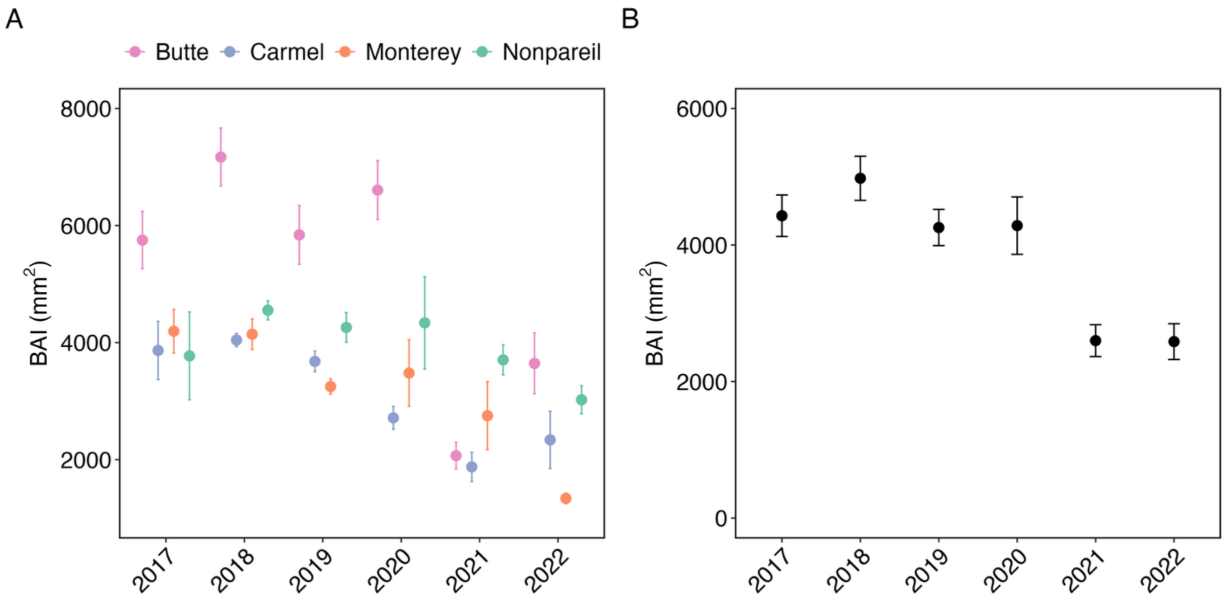

Figure S3. Basal area increment (BAI) for 2017-2022 at the (A) variety-level and (B) orchard-level. Error bars in (A) denote ± SE of the mean, and each mean was calculated from 20 trees. Error bars in (B) denote ± SE of the mean, and each mean was calculated by averaging across varieties (n=120 trees).

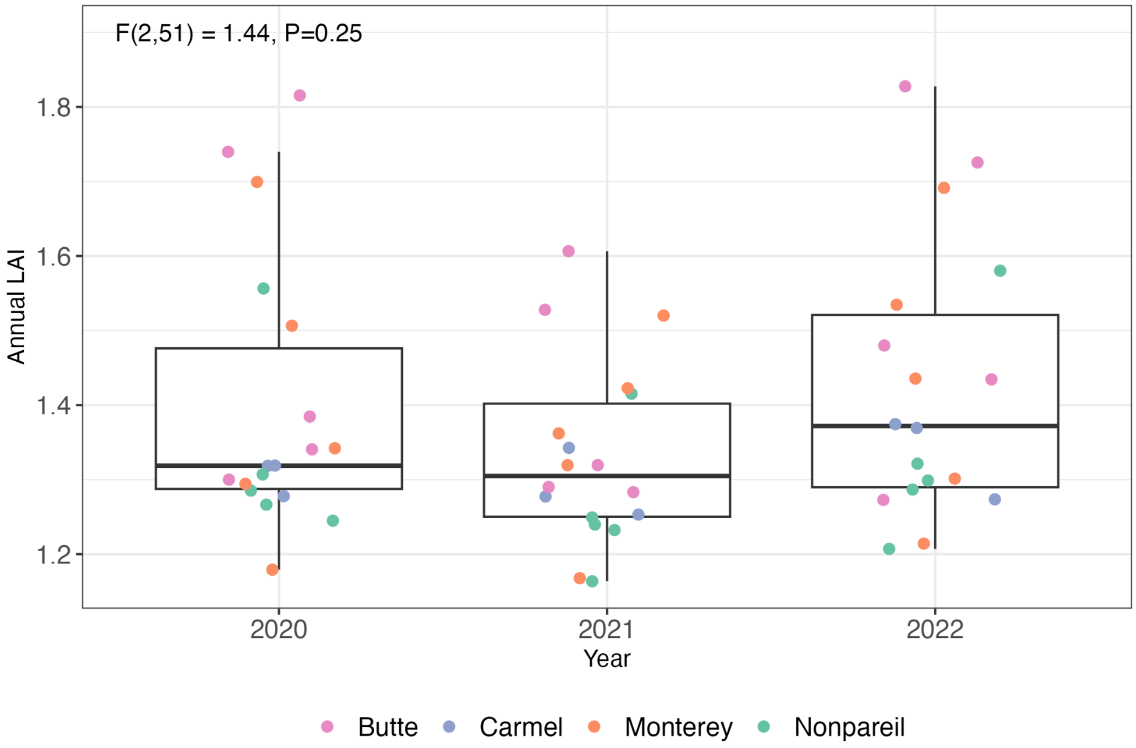

Figure S4. Comparison of leaf area index (LAI) between 2020-2022. Points represent individual trees colored by variety. LAI was derived using the CubeSat Enabled Spatio-Temporal Enhancement Method CESTEM; Houborg and McCabe, 2018), a data driven approach that combines CubeSat, Landsat 8, and MODIS observations for radiometric normalization, phenology reconstruction, and spatiotemporal enhancement, resulting in LAI estimates at 3 meter resolution. Daily LAI was then averaged across the year to obtain annual LAI for each tree. The trees selected were all trees that were cored for dendrochronology to be able to more directly consider trade-offs between C allocation to BAI versus LAI. Statistical results displayed are from one-way analysis of variance testing to analyze annual LAI among years.

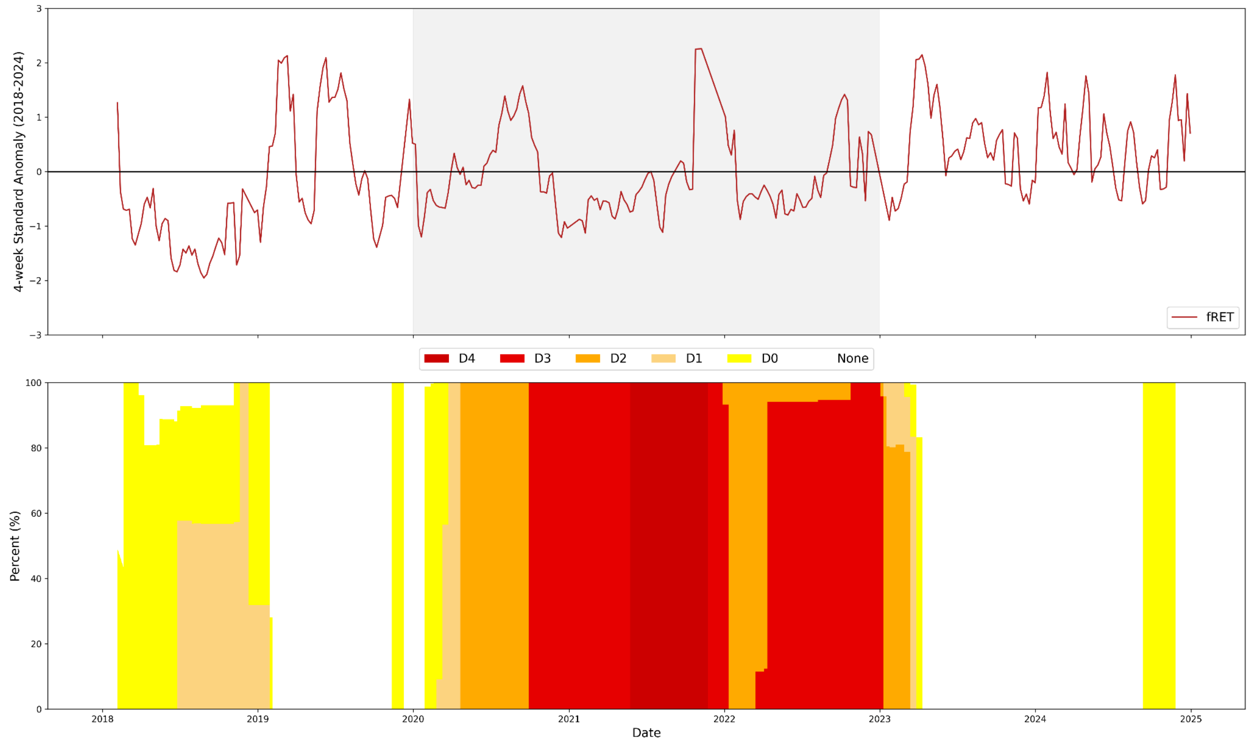

Figure S5. Time series of standard anomalies (z-scores) of the Evaporative Stress Index (ESI) and the United States Drought Monitor drought status categories for Yolo County, U.S.A. (bottom panel). The grey area overlay represents drought years 2020, 2021, and 2022. ESI anomalies were computed in 4-week moving windows stepped weekly across the 2018-2024 record. Actual Evapotranspiration and grass reference evapotranspiration were acquired via the OpenET API (https://etdata.org/api-info/) at the almond orchard location. Drought status categories are plotted as cumulative percent area (e.g., percent of county experiencing specific category).

Table S1. NSC concentration data for trees (n=120) in the study. Row and tree number identify the position of each tree in the orchard. Varieties are denoted as C = Carmel, B = Butte, M = Monterey, and NP = Nonpareil. Diameter at breast height (dbh) was measured on November 28, 2022 and is reported in cm. Branches were collected on November 1, 2021 and March 11, 2022. Stemwood was collected on November 2, 2021 and March 11, 2022. Roots were collected on November 3, 2021 and March 6, 2022. Sugar and starch concentrations for November and March are reported in mg g^{-1}.

row	tree	variety	organ	dbh	November sugar	November starch	March sugar	March starch
28	12	C	branch	19.4	30.2	29.8	38.4	5.4
28	15	C	branch	19.1	26.9	29.4	45.8	6.4
28	19	C	branch	20.1	32.6	20	37.9	4.9
28	20	C	branch	19.2	29.4	23.6	45.1	5.5
28	24	C	branch	20.3	44	28.3	35.9	3.2
28	25	C	branch	19	41	23.7	37.3	7.7
28	28	C	branch	19	31.8	28.1	40.3	5
28	31	C	branch	21.3	29.4	27.4	34.3	7.3
29	3	NP	branch	19.5	34.6	25.2	35.6	2.4
29	5	NP	branch	20.2	26.7	36.8	34.3	5.3
30	9	B	branch	22.9	31.5	25.6	33	9.1
30	10	B	branch	23	28	24.6	34.8	4.7
30	15	B	branch	26.2	31.1	21.4	35.9	6.3
30	18	B	branch	24.8	29	24.5	38.4	5.8
30	19	B	branch	24.3	23.1	25.6	44.4	10
30	21	B	branch	26.5	25.7	20.8	38.6	11.9
30	26	B	branch	23	27	26.9	37	6.4
30	28	B	branch	21.3	39.3	34.7	37	4.9
31	23	NP	branch	23.7	23.7	32.8	36.4	3.9
31	30	NP	branch	21.4	19.9	36.7	31.7	13.8
32	6	M	branch	19.2	35	30.5	33	4
32	7	M	branch	19.4	23.4	38.7	38.2	2.2
32	10	M	branch	20	29.7	33.6	41.4	2.8
32	13	M	branch	19.7	45.2	37.4	40.6	4.6
32	17	M	branch	20.1	26.9	31.7	32.1	8.2
32	18	M	branch	21.2	23.6	36.7	33.3	6.8
32	29	M	branch	21.4	24.4	30	33.9	4.4
32	30	M	branch	19.5	25.9	25.6	28.1	5.6
33	5	NP	branch	21.4	25.8	30.8	32.8	7.4
33	22	NP	branch	21.5	26.6	27.6	33	9.1
33	25	NP	branch	21.9	24.7	30.5	34.7	10.4
34	4	C	branch	17.3	26.2	24.8	33	4.7
34	5	C	branch	18.3	22.9	33.1	34.3	4.3

34	8	C	branch	18.1	22.4	28.6	37.3	4
34	10	C	branch	17.3	28.1	22.5	33.6	2.5
34	20	C	branch	19.5	24.4	35	32.8	4.7
34	22	C	branch	18.6	23.5	37.1	33.3	2.8
34	25	C	branch	18.9	27.7	36.3	41.1	7.2
35	5	NP	branch	24	22.8	38.6	29.5	6.6
35	11	NP	branch	21.5	23.5	33	31.9	6.7
35	15	NP	branch	20.8	34.2	22.4	30.3	11.6
36	5	B	branch	22.5	48.2	38.7	39.4	2.3
36	15	B	branch	22.5	41.7	35.4	41.3	1.9
36	17	B	branch	23.3	41.6	36.1	38.2	8.8
36	20	B	branch	22.9	42.3	11.7	41.7	3.2
36	24	B	branch	25.1	41.7	31.7	41.9	5.8
36	29	B	branch	23.8	37.1	37.4	36.6	7.6
36	30	B	branch	24.2	41.4	30.4	38.3	7.3
37	9	NP	branch	22.2	25.2	29.4	25.4	15
37	15	NP	branch	20.5	20	32.6	28	9.9
37	20	NP	branch	21.8	17.4	22	28.4	7
38	5	M	branch	22.2	37.6	28.7	27	6.1
38	10	M	branch	18.8	29.5	35.5	42.7	3.6
38	15	M	branch	30.2	34.4	34.3	37.5	3.9
38	27	M	branch	20.5	41.4	35.8	41.2	10.6
38	29	M	branch	22	19.3	39	39.3	4.3
38	31	M	branch	20.3	40.2	35.9	39.3	8.2
38	32	M	branch	19.7	30	39.1	33.9	2
39	5	NP	branch	21.3	23.3	31.1	23	21.2
39	10	NP	branch	22.2	31.1	25.6	31.1	24.4
39	20	NP	branch	21.5	23.1	28.9	35.4	15.5
40	8	C	branch	18.5	33.1	33.9	42.9	4.4
40	12	C	branch	20.5	25.3	31.1	35.2	6.2
40	13	C	branch	19	26.3	32.9	35.4	4.4
40	17	C	branch	19.7	26.6	36.3	42.1	7.3
40	22	C	branch	20.5	25.9	35.5	41.5	7.1
40	26	C	branch	20.4	77.3	30.5	35.5	11.6
40	32	C	branch	19.7	34.6	32	33.1	0.1
41	12	NP	branch	22	26	31.3	36	9.8
41	20	NP	branch	22.1	25	25.8	34.6	2.2
41	28	NP	branch	22.6	24.6	37.2	35.1	7.2
42	4	B	branch	22.6	35.4	28.4	44.1	6
42	5	B	branch	24.4	26.3	31.6	41.3	1.9
42	8	B	branch	23.2	28.7	33.1	47.3	3.1

42	16	B	branch	25.5	34.1	33.4	40.5	10.8
42	19	B	branch	24	36.9	35.5	49.9	1.3
42	22	B	branch	24.6	33.2	34.9	34.9	2.9
42	23	B	branch	24	29.4	32.8	39.3	6.1
43	7	NP	branch	20.7	16.6	29.4	34.2	6.1
43	12	NP	branch	19.8	27.6	27.9	36.5	6.6
43	29	NP	branch	23.2	30.9	37.3	34.7	9.8
44	3	M	branch	20.7	36.7	34.9	57.9	2.3
44	10	M	branch	19.8	37.5	37.1	40.4	11.6
44	13	M	branch	20.8	37.7	34.9	35.7	3.3
44	14	M	branch	18.1	38.8	35.3	51	7.3
44	19	M	branch	19.4	32.3	30.8	51.6	13.3
44	21	M	branch	19.8	37.2	33	43.7	7.8
44	29	M	branch	21.7	33.1	36.7	49.3	12.2
45	9	NP	branch	20.6	30.7	35	39.6	9.2
45	15	NP	branch	22.7	41.8	19.1	34.7	10.5
46	11	C	branch	18.9	33.7	33.5	37.3	11.8
46	14	C	branch	20.7	32.1	29.4	37.4	7.4
46	15	C	branch	19.5	37.5	29.2	33.2	3.6
46	18	C	branch	19.5	32.7	30.6	39.1	4
46	22	C	branch	20.5	32	33	39.2	2
46	29	C	branch	19.9	35.7	32	40	5.9
46	30	C	branch	20.9	43.6	30	36.1	13
46	31	C	branch	21.5	36.5	31.1	40.1	8.2
47	3	NP	branch	21.8	36.8	28.1	40	8.9
47	13	NP	branch	21	35.1	36.2	36.8	10.3
47	22	NP	branch	23.4	30.6	37.7	33.1	11.7
48	5	B	branch	24.1	35.9	26.9	45	8.5
48	18	B	branch	27.5	34.8	24.3	36.1	13.4
48	23	B	branch	24	40.7	21	44.4	11.4
48	25	B	branch	24.5	37.5	24.6	55	1.1
48	26	B	branch	26	26.9	34.7	42	7
48	28	B	branch	27.5	35.3	33.2	44.3	8.7
48	29	B	branch	24.6	40.3	33	52	3.4
48	31	B	branch	26	39.5	31.6	44.2	10.9
49	12	NP	branch	25.5	36.6	17.7	42.7	7.5
49	13	NP	branch	22	31.9	27.2	32.3	6.3
49	14	NP	branch	23.4	24.9	36	35.9	4.9
50	3	M	branch	19.4	37.9	33.2	37.1	13.1
50	7	M	branch	20.1	32.9	33	36.9	11.7
50	10	M	branch	20.3	52.4	35.1	41.7	8.3

50	15	M	branch	20.2	40.6	27.9	30.3	3.7
50	18	M	branch	22	45.4	26.2	39.9	8.4
50	19	M	branch	19.5	31.1	24.2	54.6	9.9
50	20	M	branch	21.9	37.3	34.9	37.9	7.4
50	27	M	branch	20.2	36.8	34.8	36.8	19.8
28	12	C	stem	19.4	19.7	38.6	16.4	21.6
28	15	C	stem	19.1	24.6	37.2	18.5	22.5
28	19	C	stem	20.1	24.4	28.3	31.7	13.3
28	20	C	stem	19.2	21.6	36.5	24.4	22.8
28	24	C	stem	20.3	28.3	31.1	24.6	20.4
28	25	C	stem	19	28.2	23.7	26.9	20.3
28	28	C	stem	19	26.6	31.7	16.4	20.7
28	31	C	stem	21.3	24.9	34.1	24.6	16.9
29	3	NP	stem	19.5	16.5	34.4	19.1	23
29	5	NP	stem	20.2	20.3	27.3	12.7	23.2
30	9	B	stem	22.9	21.3	24.5	22.7	24.3
30	10	B	stem	23	22.6	32.8	23.5	19.1
30	15	B	stem	26.2	23.1	31.6	17.5	25.6
30	18	B	stem	24.8	24.4	34.9	22.6	26.6
30	19	B	stem	24.3	21	34.6	23	23.7
30	21	B	stem	26.5	23.9	33.8	23	26.9
30	26	B	stem	23	19.2	38.5	21.6	22.3
30	28	B	stem	21.3	22.6	37.5	22.5	26.2
31	23	NP	stem	23.7	21.4	32.6	17.2	22.7
31	30	NP	stem	21.4	21.3	31.6	14.5	20.2
32	6	M	stem	19.2	13.2	37	18.1	28.5
32	7	M	stem	19.4	15.3	31.4	23.4	26.4
32	10	M	stem	20	13.4	36.8	20.4	24.4
32	13	M	stem	19.7	20.7	37.1	28.6	4.8
32	17	M	stem	20.1	23.9	36.6	15.5	29.4
32	18	M	stem	21.2	25.6	34.2	19.3	25.5
32	29	M	stem	21.4	24.4	36.7	17.8	23.8
32	30	M	stem	19.5	22.4	33.8	19.6	20
33	5	NP	stem	21.4	23	29.6	17	25
33	22	NP	stem	21.5	19.9	29.6	15.5	20.6
33	25	NP	stem	21.9	26.4	27.8	15.7	18.5
34	4	C	stem	17.3	25.6	28.9	16.4	22.6
34	5	C	stem	18.3	25.8	31.8	16.2	22.9
34	8	C	stem	18.1	25.7	27.2	22.3	18.3
34	10	C	stem	17.3	25.8	35.5	17.1	25.3
34	20	C	stem	19.5	22.1	37.1	21.2	16.3

34	22	C	stem	18.6	16	25.7	15.8	23
34	25	C	stem	18.9	25.3	31.5	17.8	22.7
35	5	NP	stem	24	18	29.7	15.1	26
35	11	NP	stem	21.5	16.8	32.7	14	26.3
35	15	NP	stem	20.8	17.8	29.5	18.2	23.7
36	5	B	stem	22.5	20.9	29.5	19.8	13
36	15	B	stem	22.5	18.3	33.5	18.8	22
36	17	B	stem	23.3	21.4	26.2	21.4	23.7
36	20	B	stem	22.9	21.5	35.7	24.5	26
36	24	B	stem	25.1	23.9	37.8	18	21.6
36	29	B	stem	23.8	19.6	38.8	22.2	29
36	30	B	stem	24.2	21.1	36.5	21.5	25.7
37	9	NP	stem	22.2	18.7	28.2	15.9	26.8
37	15	NP	stem	20.5	21.3	35.1	15.2	27.1
37	20	NP	stem	21.8	20.1	29.8	17.5	25.9
38	5	M	stem	22.2	19.1	33.9	19.2	25.5
38	10	M	stem	18.8	21	36.3	23.3	26.9
38	15	M	stem	30.2	20.9	31.7	20.9	21.5
38	27	M	stem	20.5	20	34.7	13.2	27.4
38	29	M	stem	22	21.1	36	20.4	27.5
38	31	M	stem	20.3	21.2	30	21.7	29.7
38	32	M	stem	19.7	14.9	37.3	21.4	21.6
39	5	NP	stem	21.3	16	31.5	17	24.9
39	10	NP	stem	22.2	19	31.4	18.5	26.5
39	20	NP	stem	21.5	15.1	34.1	14	20.9
40	8	C	stem	18.5	29.3	17.8	28.9	17.6
40	12	C	stem	20.5	26.1	27	23.7	26
40	13	C	stem	19	22.3	29.1	19.7	23.1
40	17	C	stem	19.7	26.2	24.2	34.9	16.2
40	22	C	stem	20.5	18.5	37.5	21	26.5
40	26	C	stem	20.4	27.2	31	22.7	24.4
40	32	C	stem	19.7	25.8	27.8	22.4	24.2
41	12	NP	stem	22	21.8	27.8	19.2	25.9
41	20	NP	stem	22.1	20	28.9	21.5	21.3
41	28	NP	stem	22.6	22.1	32	21.4	23.2
42	4	B	stem	22.6	22	31	20.3	24.5
42	5	B	stem	24.4	23.1	32.7	18.5	21.8
42	8	B	stem	23.2	25.3	27.3	21.4	19.6
42	16	B	stem	25.5	21.2	32.2	23.1	21.6
42	19	B	stem	24	23.3	27	19.7	22.4
42	22	B	stem	24.6	17.3	36	23	24.3

42	23	B	stem	24	21	28	23.6	23.2
43	7	NP	stem	20.7	18.4	22.9	19.9	25.2
43	12	NP	stem	19.8	20.9	31.7	18.4	24
43	29	NP	stem	23.2	27.8	22.2	19.5	24
44	3	M	stem	20.7	23.4	19.3	24.6	17.8
44	10	M	stem	19.8	25.3	36.7	27	23.8
44	13	M	stem	20.8	25.2	30.5	27.2	23.4
44	14	M	stem	18.1	22.9	27.5	25.6	9.9
44	19	M	stem	19.4	27.4	34.4	24.6	21
44	21	M	stem	19.8	28.8	38.3	19	18.1
44	29	M	stem	21.7	30.3	31.5	27.2	27.2
45	9	NP	stem	20.6	19.4	29.3	24.1	23.4
45	15	NP	stem	22.7	23.2	26.7	21.4	25.1
46	11	C	stem	18.9	22.8	26.9	19.6	19.6
46	14	C	stem	20.7	27.8	24.3	28.4	13.8
46	15	C	stem	19.5	26.9	26	16.4	22.5
46	18	C	stem	19.5	26.6	1.7	40.8	22.1
46	22	C	stem	20.5	35.4	26.1	34.5	25.5
46	29	C	stem	19.9	18	19.4	32	24.9
46	30	C	stem	20.9	27.2	25.6	29.4	21.3
46	31	C	stem	21.5	27.2	15.7	26.8	9
47	3	NP	stem	21.8	27.1	27.4	14.1	14
47	13	NP	stem	21	16.3	29.3	20.1	24
47	22	NP	stem	23.4	27.4	12.3	24.8	19.5
48	5	B	stem	24.1	33.4	30.5	25.1	19.4
48	18	B	stem	27.5	28.1	10.2	36.7	16.1
48	23	B	stem	24	46.1	23.5	32.6	19.3
48	25	B	stem	24.5	16	30.3	25.8	22.6
48	26	B	stem	26	28.4	28.8	24.6	22.4
48	28	B	stem	27.5	28.7	33.1	25.4	20.7
48	29	B	stem	24.6	30.4	35.9	21.8	22.7
48	31	B	stem	26	19.8	29.8	27.9	20.7
49	12	NP	stem	25.5	26.3	24.1	27.2	36.7
49	13	NP	stem	22	27.6	27.6	16.6	11.2
49	14	NP	stem	23.4	20.2	25.5	22.9	2.4
50	3	M	stem	19.4	34	29.1	24.4	23.2
50	7	M	stem	20.1	24.1	31.9	24.7	13.3
50	10	M	stem	20.3	25.9	35	23.6	24.4
50	15	M	stem	20.2	34.5	30.5	19.7	21.7
50	18	M	stem	22	27.9	30.9	20.7	31.2
50	19	M	stem	19.5	32.3	29	22.5	23.3

50	20	M	stem	21.9	37	28.7	18	27.6
50	27	M	stem	20.2	24.4	38	21.6	24.7
28	12	C	root	19.4	19.9	79.2	15.2	72.1
28	15	C	root	19.1	17.9	69.7	22.6	33.9
28	19	C	root	20.1	12.3	75.5	15.9	60.4
28	20	C	root	19.2	17.5	79	16.4	52.1
28	24	C	root	20.3	16.6	61.3	15.2	57.5
28	25	C	root	19	20.5	75.9	23.9	50.8
28	28	C	root	19	21.7	67.4	16.8	46.8
28	31	C	root	21.3	21	63.6	17.8	72.6
29	3	NP	root	19.5	16.3	33.6	17.4	44.3
29	5	NP	root	20.2	18.1	94	17.9	34.1
30	9	B	root	22.9	12.3	92.6	16.2	34.7
30	10	B	root	23	9.6	51.1	20.9	38.2
30	15	B	root	26.2	14.9	73.2	15.7	10.2
30	18	B	root	24.8	19.3	81.6	18.3	46.1
30	19	B	root	24.3	20	70.5	13.1	32.9
30	21	B	root	26.5	13	65.8	14.2	48.4
30	26	B	root	23	19.2	58.3	16.8	49.7
30	28	B	root	21.3	14.9	60.5	14.7	48.4
31	23	NP	root	23.7	17.9	83.1	16.4	39.2
31	30	NP	root	21.4	23.8	74.3	9.3	21.3
32	6	M	root	19.2	15.3	96.6	20.3	72.2
32	7	M	root	19.4	18.8	84.1	16	13.9
32	10	M	root	20	17.1	71.3	14.7	50.4
32	13	M	root	19.7	19.4	64.2	19.7	15.1
32	17	M	root	20.1	16.6	73.1	14.7	57.9
32	18	M	root	21.2	17.2	88.8	17.3	72.1
32	29	M	root	21.4	17.8	47.9	11	9.2
32	30	M	root	19.5	15.6	83.4	18	63.2
33	5	NP	root	21.4	22.8	69	17.7	55
33	22	NP	root	21.5	15.6	93.7	9	59.9
33	25	NP	root	21.9	17.7	77.7	16.2	47.7
34	4	C	root	17.3	17.5	123.6	17.9	67.2
34	5	C	root	18.3	22.9	58.6	18.3	52.1
34	8	C	root	18.1	15.5	142	15.8	75.8
34	10	C	root	17.3	20.6	75.2	20.9	46.8
34	20	C	root	19.5	15.4	51.9	14.6	68
34	22	C	root	18.6	16.2	78.3	15.1	45.4
34	25	C	root	18.9	17.8	90.7	9.8	38.2
35	5	NP	root	24	17.6	49.4	20.9	34.4

35	11	NP	root	21.5	12.5	77.2	17.3	13.4
35	15	NP	root	20.8	18.3	89.4	19.1	26.2
36	5	B	root	22.5	15.5	49.4	17.9	44.8
36	15	B	root	22.5	16.4	91.2	13.6	44.2
36	17	B	root	23.3	15.7	76.8	15.1	54.5
36	20	B	root	22.9	15.3	69.5	16.7	53.7
36	24	B	root	25.1	12.4	68.8	13.8	44.6
36	29	B	root	23.8	17.4	93.1	16.1	63.1
36	30	B	root	24.2	15.9	83.6	24.6	66.4
37	9	NP	root	22.2	14	83.4	18.5	51.6
37	15	NP	root	20.5	19.2	105.7	24.4	43.8
37	20	NP	root	21.8	16.3	77.8	16.4	57.3
38	5	M	root	22.2	18.1	80.6	11.3	66.4
38	10	M	root	18.8	17.8	44.2	16	12.5
38	15	M	root	30.2	19.1	84.5	20.4	53.5
38	27	M	root	20.5	24.8	82.8	25.3	17
38	29	M	root	22	12.5	68.8	19	63.5
38	31	M	root	20.3	11.5	96.7	22.8	42
38	32	M	root	19.7	16.5	75.2	20.6	57.2
39	5	NP	root	21.3	16.3	65.8	16.4	47.9
39	10	NP	root	22.2	18.9	86.1	20.1	27.8
39	20	NP	root	21.5	19.7	48.3	18.6	15.8
40	8	C	root	18.5	18.7	103.3	16.8	62.3
40	12	C	root	20.5	19.6	89.3	16	53.8
40	13	C	root	19	11.8	74.6	22	7.6
40	17	C	root	19.7	19.1	79.9	19.1	57.5
40	22	C	root	20.5	17.9	40.9	19	11.2
40	26	C	root	20.4	19.5	63.8	24.2	60.8
40	32	C	root	19.7	16.3	87.9	17	67.9
41	12	NP	root	22	21.7	72.9	19.7	48.1
41	20	NP	root	22.1	19	49.3	17.1	29.9
41	28	NP	root	22.6	22.8	58.7	17	65.3
42	4	B	root	22.6	16.3	65.2	14.6	32.1
42	5	B	root	24.4	17.7	49.6	19.6	31.2
42	8	B	root	23.2	18.5	69.5	22.5	16.5
42	16	B	root	25.5	16.7	58.4	17.2	115.1
42	19	B	root	24	11.5	50.3	16.1	46.8
42	22	B	root	24.6	15.2	60.3	15.5	45.3
42	23	B	root	24	17.6	82.5	17.7	18
43	7	NP	root	20.7	17.8	60.2	14.7	56.3
43	12	NP	root	19.8	16.1	79.3	23.2	25

43	29	NP	root	23.2	14.7	52.3	17.2	42.9
44	3	M	root	20.7	14	70.2	17.8	59.5
44	10	M	root	19.8	18.8	29.4	20.7	17.6
44	13	M	root	20.8	19.4	36.5	18.2	47
44	14	M	root	18.1	24.2	62.2	26.2	43.2
44	19	M	root	19.4	19	55.2	21.1	18.3
44	21	M	root	19.8	20.9	64.5	17.7	58.6
44	29	M	root	21.7	14.7	55.2	16.9	53.8
45	9	NP	root	20.6	20.4	76.6	17.6	58.9
45	15	NP	root	22.7	18.4	56.3	23.9	11.4
46	11	C	root	18.9	17.6	66	20.8	63.1
46	14	C	root	20.7	17.7	64.8	23.4	8.6
46	15	C	root	19.5	18.4	39.6	11.7	48.9
46	18	C	root	19.5	17.6	43.7	28.9	47.1
46	22	C	root	20.5	18	67	17.4	64.9
46	29	C	root	19.9	17.2	64.3	13.7	47.2
46	30	C	root	20.9	18.5	28.6	20.4	14.1
46	31	C	root	21.5	17.7	53.3	25.1	40.2
47	3	NP	root	21.8	12.2	45.5	15.7	53.1
47	13	NP	root	21	11.9	80.2	15	21.7
47	22	NP	root	23.4	15.7	49	17.3	18.9
48	5	B	root	24.1	16.9	40.2	16.5	23.2
48	18	B	root	27.5	15.4	63.5	25.8	92.6
48	23	B	root	24	19.4	54.3	16.4	60.7
48	25	B	root	24.5	18.5	61.3	13.8	57.5
48	26	B	root	26	18.3	67.1	18.7	26.6
48	28	B	root	27.5	21.3	38.5	20.7	19.2
48	29	B	root	24.6	15.9	79.5	17.4	72.6
48	31	B	root	26	17.7	60.7	16.6	70.3
49	12	NP	root	25.5	13.3	50.3	18.2	30.9
49	13	NP	root	22	20	62.4	19.1	25.3
49	14	NP	root	23.4	18.7	77	15.8	47.6
50	3	M	root	19.4	15.3	56	14.7	58.9
50	7	M	root	20.1	13.3	55.8	12.4	25.8
50	10	M	root	20.3	18.2	44.3	15.7	18.1
50	15	M	root	20.2	25.1	67.3	21	13.1
50	18	M	root	22	15.9	66.6	17.2	21.4
50	19	M	root	19.5	17.3	61.4	15.2	44.9
50	20	M	root	21.9	18.6	41.9	23.4	16.3
50	27	M	root	20.2	15.7	64.4	19.3	28

Table S2. Results of one-sample t-tests comparing the difference in sugar, starch, or total NSC concentrations (March 2022 - November 2021) (mg g^{-1}) for each variety and organ to a null hypothesis of 0. The table contains output values for the mean, t-value, degrees of freedom (df), p-value, and significance symbol (symbol). P-values are displayed in Figure 1 as follows: <0.0001 = ***, <0.01 = **, <0.05 = *, and >0.05 = ns.

variety	type	organ	mean	t-value	df	p-value	symbol
Butte	sugar	branch	6.4	4.7933	29	4.505e-05	***
Carmel	sugar	branch	5.2	2.5425	29	0.01661	*
Monterey	sugar	branch	5.1	2.907	29	0.006926	**
Nonpareil	sugar	branch	6.2	6.1776	29	9.796e-07	***
Butte	starch	branch	-23.1	-16.278	29	4.003e-16	***
Carmel	starch	branch	-24.5	-26.963	29	2.2e-16	***
Monterey	starch	branch	-26.2	-23.924	29	2.2e-16	***
Nonpareil	starch	branch	-21.0	-15.062	29	3.02e-15	***
Butte	total	branch	-16.6	-8.1201	29	5.919e-09	***
Carmel	total	branch	-19.4	-10.036	29	6.085e-11	***
Monterey	total	branch	-21.2	-10.949	29	8.108e-12	***
Nonpareil	total	branch	-14.8	-11.796	29	1.372e-12	***
Butte	sugar	stem	-0.56	-0.59454	29	0.5568	ns
Carmel	sugar	stem	-1.3	-1.1732	29	0.2502	ns
Monterey	sugar	stem	-2.2	-1.8575	29	0.07342	ns
Nonpareil	sugar	stem	-2.7	-3.3526	29	0.00224	**
Butte	starch	stem	-8.9	-10.011	29	6.444e-11	***
Carmel	starch	stem	-7.1	-5.0182	29	2.411e-05	***
Monterey	starch	stem	-10.0	-8.2039	29	4.796e-09	***
Nonpareil	starch	stem	-6.0	-4.8674	29	3.667e-05	***
Butte	total	stem	-9.4	-6.7475	29	2.097e-07	***
Carmel	total	stem	-8.4	-3.8949	29	0.0005319	**
Monterey	total	stem	-12.3	-8.8816	29	9.056e-10	***
Nonpareil	total	stem	-8.8	-5.5294	29	5.832e-06	***
Butte	sugar	root	0.9	1.2942	29	0.2058	ns
Carmel	sugar	root	0.4	0.50595	29	0.6167	ns
Monterey	sugar	root	0.5	0.76058	29	0.453	ns
Nonpareil	sugar	root	-0.02	-0.024637	29	0.9805	ns
Butte	starch	root	-19.3	-4.0833	29	0.0003189	**
Carmel	starch	root	-22.1	-5.4377	29	7.519e-06	***
Monterey	starch	root	-25.5	-7.003	29	1.062e-07	***
Nonpareil	starch	root	-30.8	-7.7805	29	1.401e-08	***
Butte	total	root	-18.4	-3.753	29	0.000779	**
Carmel	total	root	-21.7	-5.3708	29	9.054e-06	***
Monterey	total	root	-25.5	-7.003	29	1.062e-07	***
Nonpareil	total	root	-30.8	-7.7805	29	1.401e-08	***

Table S3. Results of one-way ANOVA testing to analyze the difference in sugar, starch, or total NSC concentrations (March 2022 - November 2021) (mg g^{-1}) among varieties for each NSC type and organ. When significant, differences between pairs of means were evaluated with Tukey's honest significant difference (HSD), α = 0.05, and uppercase letters in Figure 1 indicate significance of differences among varieties. The table contains output values for the F-value, degrees of freedom (df; variety, residuals), p-value, and Tukey's significance letters for each variety when applicable.

type	organ	F-value	df	p-value	Tukey's significance letters for			
					Butte	Carmel	Monterey	Nonpareil
sugar	branch	0.1967	(3,116)	0.8984				
starch	branch	3.3612	(3,116)	0.0212	AB	AB	A	B
total	branch	2.4454	(3,116)	0.0675				
sugar	stem	0.8565	(3,116)	0.4659				
starch	stem	2.1859	(3,116)	0.0934				
total	stem	1.1074	(3,116)	0.3491				
sugar	root	0.2588	(3,116)	0.855				
starch	root	1.4639	(3,116)	0.2281				
total	root	1.6432	(3,116)	0.1833				

Table S4. Results of one-way ANOVA testing to analyze the difference in total NSC concentrations (March 2022 - November 2021) (mg g^{-1}) among organs. When significant, differences between pairs of means were evaluated with Tukey's honest significant difference (HSD), α = 0.05. The table contains output values for the F-value, degrees of freedom (df; variety, residuals), p-value, and Tukey's significance letters for each organ when applicable.

F-value	df	p-value	Tukey's significance letters for		
			branch	stem	root
26.254	(2,357)	2.304e-11	A	B	C

Table S5. Summary output from correlation analyses using function cor.test() in R. Strength of association was evaluated using Pearson's correlation at $\alpha = 0.05$. Results are displayed in Figure 2, Figure 3, and Figure 4.

Variable 1	Variable 2	Correlation coefficient r	t-value (df)	p-value
**Yield**<br>Units: Net weight (kg/hectare/yr)<br>Scope: variety (yield for each variety across the orchard)<br>Time: annually 2017-2022	**Stem growth**<br>Units: BAI (mm^2/yr)<br>Scope: variety (BAI for each variety based on 5 trees each)<br>Time: annually 2017-2022	-0.39	-1.9965(22)	0.0584
**Yield**<br>Units: Net weight (kg/hectare/yr)<br>Scope: orchard<br>Time: 2016-2022	**Stem growth**<br>Units: RWI (unitless)<br>Scope: orchard<br>Time: 2016-2022	-0.55	-1.4603(5)	0.204
**Branch total NSC**<br>Units: total NSC (mg g^{-1})<br>Scope: variety (NSC for each variety based on 30 trees each)<br><br>Time: entering dormancy in November 2021	**Yield**<br><br>Units: Net weight (kg/hectare/yr)<br>Scope: variety (yield for each variety across the orchard)<br>Time: 2022	0.96	4.6597(2)	0.0431
**Stem total NSC**<br>Units: total NSC (mg g^{-1})<br>Scope: variety (NSC for each variety based on 30 trees each)<br>Time: entering dormancy in November 2021	**Yield**<br>Units: Net weight (kg/hectare/yr)<br>Scope: variety (yield for each variety across the orchard)<br>Time: 2022	0.92	3.3587(2)	0.07837

**Stem total NSC**<br>Units: total NSC (mg g^{-1})<br>Scope: 20 trees<br>Time: entering dormancy in November 2021	**Stem growth**<br>Units: BAI (mm^2)<br>Scope: 20 trees<br>Time: 2022	-0.55	-2.7967(18)	0.01192
**Stem sugar**<br>Units: sugar (mg g^{-1})<br>Scope: 20 trees<br>Time: exiting dormancy in March 2022	**Stem growth**<br>Units: BAI (mm^2)<br>Scope: 20 trees<br>Time: 2022	0.52	2.5508(18)	0.02007
**Stem total NSC**<br><br>Units: total NSC (mg g^{-1})<br>Scope: 120 trees<br>Time: entering dormancy in November 2021	**ΔStem total NSC**<br>Units: Δ total NSC (mg g^{-1})<br>Scope: 120 trees<br>Time: March 2022 minus November 2021	-0.74	-12.047<br>(118)	2.2e-16

Methods S1. Allometric scaling from NSC concentrations to whole-tree reserves

We used the parameters from Jenkins *et al*. (2003) and the updated parameters in Chojnacky *et al*. (2014) to estimate the biomass components of the almond trees in our study (n=120 trees) and then used these biomass estimates to scale NSC concentrations up to whole-tree reserves. In Jenkins *et al*. (2003), we used the parameters for the mixed hardwood species group to estimate total aboveground biomass as a function of diameter at breast height for each almond tree. We then estimated the fraction corresponding to branches, stemwood, and coarse roots and then estimated the biomass of each component. We then paired total NSC concentrations with the biomass estimate of each component, multiplied them together, and then summed over all components to determine the whole-tree reserves for each tree.

In Chojnacky *et al.* (2014), we used the parameters for the hardwood group and taxa Rosaceae to estimate total aboveground biomass as a function of diameter at breast height for each almond tree. We then estimated the coarse root biomass using the parameters for *Prunus*. We summed total NSC concentrations in the branches and stems to get a total NSC concentration for aboveground organs. We then paired total NSC concentrations for aboveground and belowground organs with the biomass estimate for each, multiplied them together, and then summed over both components to determine the whole-tree reserves for each tree.
